# Supplementary material for: The relationship between management practices and health facility performance: Evidence from low-resource, community-based facilities providing HIV services to key populations
Source: PLoS One. 2025 Aug 28;20(8):e0330300. doi: 10.1371/journal.pone.0330300 (PMC12393696; doi:10.1371/journal.pone.0330300)
Supplement: S1 Table — aAs defined in [18]. All items coded as 1 = Yes, 0 = No. (PDF) [file pone.0330300.s001.pdf]

Management dimension	Meaning [a]	Number of items included	Activities carried out at the DIC during 2018- 2019
**Target setting**	Setting appropriate targets, tracking correct outcomes, and ensuring that targets and outcomes align.	7	1. DIC-specific goals were set every year, for example number of individuals reached or testing rates
			2. The DIC had goals related to the spending plan (budget).
			3. The DIC set goals related to community involvement.
			4. The DIC set goals related to marketing and demand generation.
			5. The DIC set goals for individual staff members.
			6. The DIC set goals at the team level.
			7. A timeline was made and updated to reach the staff goals and targets.
**Performance monitoring**	Describes the collection and analysis of data to understand an organization's performance and identify opportunities for improvement	18	**Activities carried out at the DIC during 2018- 2019**
			1. The DIC was required to inform external entities about facility performance.
			2. The DIC was required to inform external entities about staff performance.
			3. The DIC informed external entities about their performance on meeting supply targets
			4. The DIC informed external entities about their performance on meeting budgets and expenditures
			5. The DIC, procedures were sent/shown to an external entity.
			6. The DIC organized internal meetings to report DIC performance.
			7. The in-country office supervised the frequency and content of meetings held at the DIC
			8. The DIC was required to send evidence of meetings and agreements related to service provision
			**External entity evaluation**
			1. External entity evaluate the DIC in terms of: Stock of supplies
			2. External entity evaluate the DIC in terms of: Staff performance
			3. External entity evaluate the DIC in terms of: Budget and expenditures
			4. External entity evaluate the DIC in terms of: Quality of care
			5. External entity evaluate the DIC in terms of: DIC layout
			**Periodic internal review evaluation**
			1. The DIC engage in periodic internal review evaluation of: Stock of supplies
			2. The DIC engage in periodic internal review evaluation of: Staff performance

			3. The DIC engage in periodic internal review evaluation of: Budget and expenditures
			4. The DIC engage in periodic internal review evaluation of: Quality of care
			5. The DIC engage in periodic internal review evaluation of: DIC layout
**People management**	It includes the various activities related to hiring, retaining, and rewarding high performance and addressing underperformance.	20	**Structures or activities that were normally presented or carried out at the DIC during 2018/2019**
			1. The DIC evaluates the performance of its operational staff.
			2. Staff members receive incentives or rewards to recognize their performance
			3. Staff members receive sanctions for poor performance.
			4. Staff that is not engaged or is partially engaged, receives the full salary at the month end
			**Types of incentives or rewards implemented among staff during 2018/2019**
			1. Time off
			2. Verbal recognition
			3. Written recognition / certificates
			4. Monetary bonuses
			5. Subsidized trainings/courses
			6. Preferred schedule
			7. Commodities (e.g., food basket)
			**Types of incentives sanctions or rewards were implemented among staff during 2018/2019**
			1. Verbal warning
			2. Written warning
			3. Forced leave or relocation
			4. Pay reduction
			5. Become ineligible for monetary bonuses (e.g., per diem, punctuality bonus)
			6. Less desirable schedule
			**Training**
			1. DIC manager or any of the employees of the facility attended any training
			2. The facility have training plans for clinical staff
			3. During 2018-2019, the DIC manager was trained in topics related to general management
**Operations managemen t**	It refers to the extent to which processes in the organization are standardized and operations are continuously improved	11	**Structures or activities were normally present / carried out at the DIC during 2018-2019**
			1. The DIC has an established schedule to perform organization-related activities, such as sorting, labelling, and filing documents (e.g., patient records, MoH registers)
			2. The DIC has a dedicated space to store lab inputs (e.g., Rapid Diagnostic Tests (RDTs), lab reagents)

			3. The DIC has a dedicated space to store drugs
			4. Medical supplies are stored and organized, as soon as they arrive
			5. There is a documented process that describes how to manage drug stock, RDTs and other lab inputs
			6. The DIC does not feel crowded to walk by
			7. The DIC usually operates in special schedules, to reach key populations (e.g., night shifts, weekends)
			8. When the workload is heavy, the DIC employs locum nurses from other health facilities (e.g., government hospitals)
			9. Procedures for staff are documented in a manual and easily accessible for consultation (SOPs)
			10. Procedures are posted on the walls or blackboards of the DIC
			11. Reminders are posted in a special place of the DIC (e.g., color-coded biohazard disposal reminders)
**Financial management**	It describes the budgeting and financial accounting of revenues and expenses to ensure the smooth operations of an organization	7	**Structures or activities were normally present / carried out at the DIC during 2018-2019**
			1. The DIC handle financial resources directly, e.g., revenue for providing health services, direct donations for purchasing inputs/paying utilities
			2. The DIC provides a LINKAGES financial report every month
			3. The DIC does a financial audit for itself every year on total facilities revenues and expenditures
			4. Someone outside of the DIC (externals) come to do a financial audit every year on total DIC revenues and expenditures
			5. The DIC elaborates spending plans (budget) each year
			6. All payments are done through the bank / mobile money
			7. The DIC has a dedicated bank account to manage DIC expenses
**Community engagement**	It includes the development and management of relationships with community members through community outreach, involvement of community leaders, and activities to ensure customer trust and satisfaction	4	1. During 2018-2019, the DIC had a DIC committee / governing board
			2. During 2018-2019, members of the community involved in the DIC committee / governing board
			3. During 2018-2019, members of the community participated in the budget decision making process
			4. During 2018-2019, members of the community participated in expenditures decisions?
