# Supplementary material for: The relationship between management practices and health facility performance: Evidence from low-resource, community-based facilities providing HIV services to key populations
Source: PLoS One. 2025 Aug 28;20(8):e0330300. doi: 10.1371/journal.pone.0330300 (PMC12393696; doi:10.1371/journal.pone.0330300)
Supplement: S1 File — (PDF) [file pone.0330300.s002.pdf]

For simplicity, let us assume there are only two management dimensions in Equation (3), $\widehat{m}_i^1$, $\widehat{m}_i^2$, and that R^2 is the indicator of interest to be decomposed, in our case, the relative explained variance in performance. The marginal contribution of $\widehat{m}_i^1$ is computed by first estimating the indicator of interest, $R^2_{\widehat{m}_i^1}$, when only $\widehat{m}_i^1$ is included. Second, we re-estimate, R^2 when both covariates are included, $R^2_{\widehat{m}_i^1,\widehat{m}_i^2}$. Third, the marginal contribution of $\widehat{m}_i^1$ is obtained by subtracting the $R^2_{\widehat{m}_i^1}$ value from the $R^2_{\widehat{m}_i^1,\widehat{m}_i^2}$ value. The Shapley contribution for $\widehat{m}_i^1$ is then given by averaging across all potential permutations. The same process applies to getting the marginal contribution of $\widehat{m}_i^2$. Adding more management dimensions implies higher computational complexity as all possible permutations must be estimated, e.g., 2^m, where *m* represents the number of management dimensions.
