# Supplementary figures and images for: The relationship between management practices and health facility performance: Evidence from low-resource, community-based facilities providing HIV services to key populations
Source: PLoS One. 2025 Aug 28;20(8):e0330300. doi: 10.1371/journal.pone.0330300 (PMC12393696; doi:10.1371/journal.pone.0330300)

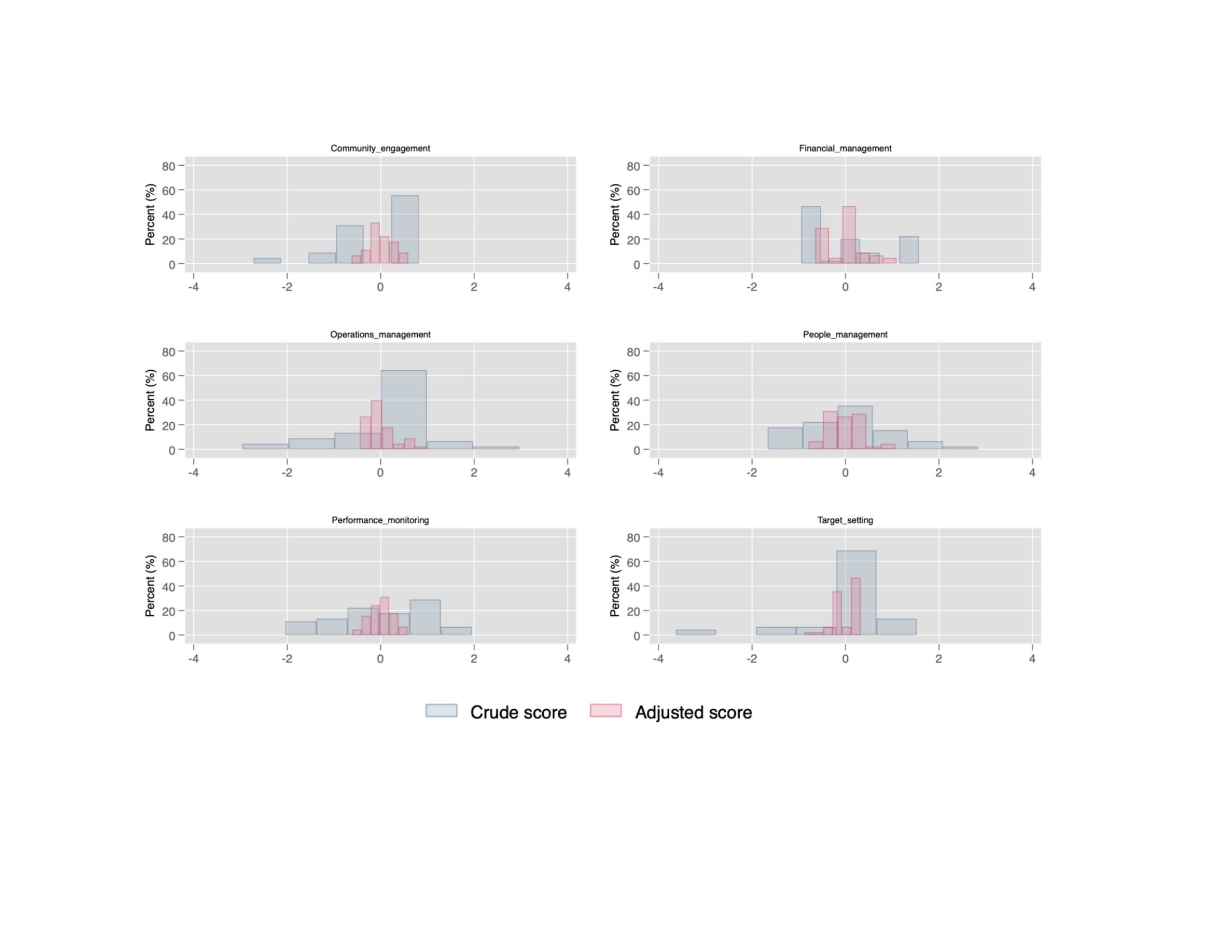

Supplement: S1 Fig — Adjusted management practices for contextual characteristics to control for differences in scores across CBOs. (TIF) [file pone.0330300.s003.tif]
