# Supplementary material for: The relationship between management practices and health facility performance: Evidence from low-resource, community-based facilities providing HIV services to key populations
Source: PLoS One. 2025 Aug 28;20(8):e0330300. doi: 10.1371/journal.pone.0330300 (PMC12393696; doi:10.1371/journal.pone.0330300)
Supplement: Attachment — Submitted filename: revision comments.pdf [file pone.0330300.s004.pdf]

1. **General comment:** I found the work highly insightful for publication however I have suggested some minor recommendations to make the work stronger
2. **Sectional comments**
   a. **Abstract-** this is well written but there is need to revise the conclusion to provide a brief summary of the key findings, potential implications, and the way forward.
   b. **Background:** This text is also well composed; however, an additional paragraph following line 85 on page 4, which justifies the selection of the two countries supported by statistics aligned with the study's objectives, would be great.
   c. **Methods**- this ok but the authors may consider adding an introduction of study design between line 99-100. Despite the citation provided to the used technique elsewhere, a brief description of the sampling and selection criteria used to choose the DIC is necessary under line 117 on page 6. Also provide rationalize linking the current study to the phase 1 and 2. In page 10, line 204-205; the authors may anchor the application of Shapley decomposition approach with a theory.
   d. **Results**- the statistics used are sound but values are not provided in the text. The authors need to include the correlation coefficient, DF and p value appropriately in variables in line 261-274: statistics values in the text (%) in line 279-287 and p-values in 295-307. Additionally, the CHO characteristics may be enriched with country level analysis that may reveal in-depth dimension essential to policy makers in each country. Figure 1 appears to be missing in line 255.
   e. **Discussion-** this is well written but the authors may provide a proposition to explain their finding in line 317. Also state the limitation for application of Shapley decomposition.
   f. **Conclusion**: revise the conclusion to provide key finding, potential implication and scale-able management way forward

General rubrics

- The authors may consider splitting long sentences into in line 49-51, 51-54, 59-61, 76-79, 82-85, 217-221
- Delete the word **of** in 225.
